# Extracts from Foreign Journals

**Published:** 1895-12

**Authors:** 


### EXTRACTS FROM FOREIGN JOURNALS.

Moret has noted echinococci in three asses and one horse. Twelve cysts were found, all of them in the lung. Some were calcified, one was filled with pus. In eleven the hydatid membrane was present, but scolices were found only twice; in the others only fluid was present.

Dlugay fed two small dogs with the meat of a hog containing trichinæ, and both became ill. One, which was very old, recovered; in the other restlessness appeared in the third week after ingestion of the meat; a little later the dog was unable to walk, had bloody stools and frequent spasms. Still later, the hind parts became absolutely paralyzed. The temperature was 39.4° C. There was a purulent discharge from the eyes. The dog was then killed, and at the autopsy numerous trichinæ were found in the muscles, but there were no other lesions of importance.

Ruser has found œstrus larvæ in the spinal canal in cattle.

Cardiot and Roger noted in a hen a hemispherical sensitive swelling in the right periorbital region. The eyelids were closed by the tumor, though the eye was uninjured. The swelling was laid open, and a cheesy mass was evacuated, which was found to contain many tubercle bacilli.

They report, also, the case of a parrot in which there was a swelling the size of a nut under the wing, in the region of the carpal articulation. The tumor consisted of fibrous tissue containing cheesy matter in the middle. In the fibrous portion there were tubercles with many tubercle bacilli, and in the cheesy mass there were a few bacilli. Two guinea-pigs were inoculated with this matter, and both died in forty-five and forty-eight days, respectively, from disseminated tuberculosis. This, of course, is unusual, for aviary tuberculosis usually causes only local lesions in guinea-pigs. A hen was also inoculated from the same case, but remained healthy, and when killed at the end of two months showed no pathological changes.

Tubercles of the Lungs not due to Farcy. In the horse's lungs there are, not rarely, tubercles, probably embolic, which have given cause for some confusion with farcy. In the earliest stages they resemble frog's eggs, later they have a tough, connective-tissue wall and become cheesy or calcified. The size varies up to the size of a pea. Usually pretty numerous.

They are distinguishable from farcy tubercles, to the naked eye, by the absence of a red aurelia, by the tendency to become calcified, by the uniform consistency of the nodules, by the absence of further evidences of farcy, and by the normal conditions of the bronchial glands. One observer has found a fungous growth in the nodule, another has found distomata, and a third echinococci. This is a condition that is questionable.

Nodules are said to be found in Pomerania, in over 70 per cent. of the horses which come to autopsy.

---

The following item, which has been going the rounds of the daily journals, loses its unique character when one learns that the school alluded to has never graduated a member of the feminine sex: Miss Edith Oakey, a graduate of the Toronto Veterinary College, now in active practice at Sandford, Ohio, is the only woman in America with a veterinary diploma. She gives special attention to bovine practice, and enjoys a lucrative practice, delegating to male assistants much of the laborious parts of her work.

